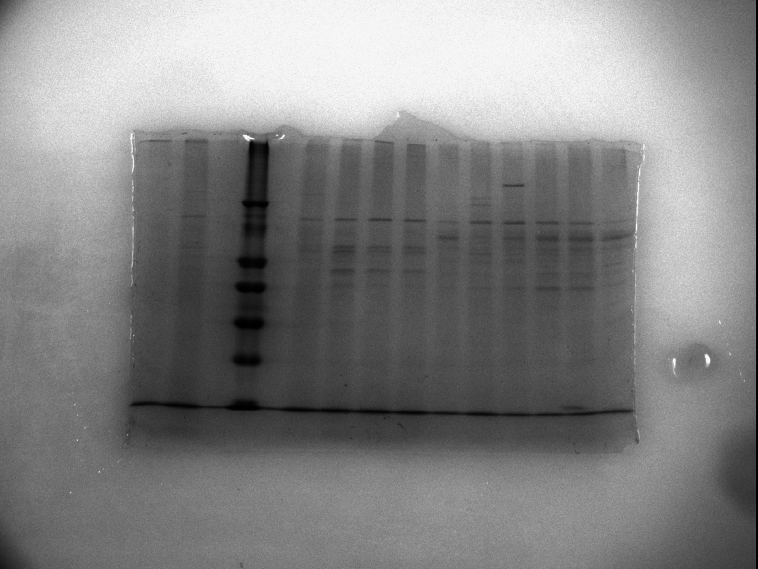# Improving protein extraction and peptide production from *Chlorella vulgaris* using combined mechanical/physical and enzymatic pre-treatments

**DOI:** 10.1016/j.heliyon.2024.e32704

**Published:** 2024-06-07

**Authors:** Mónica Mendes Costa, Maria Pinheiro Spínola, Victor Diogo Alves, José António Mestre Prates

**Affiliations:** a CIISA - Centro de Investigação Interdisciplinar em Sanidade Animal, Faculdade de Medicina Veterinária, Universidade de Lisboa, Av. da Universidade Técnica, 1300-477, Lisboa, Portugal; b Associate Laboratory for Animal and Veterinary Sciences (AL4AnimalS), Faculdade de Medicina Veterinária, Universidade de Lisboa, Av. da Universidade Técnica, 1300-477, Lisboa, Portugal

**Keywords:** *Chlorella vulgaris*, Microalgae, Mechanical pre-treatments, Enzymatic pre-treatments, Protein solubility

## Abstract

*Chlorella vulgaris* is a microalga rich in proteins with potential applications in food and feed industries. However, the presence of a cellulose-containing cell wall, which is a major barrier to protein extraction, together with fibroproteinaceous complexes, limits the bioaccessibility of nutritional and bioactive proteins and peptides from *C. vulgaris* biomass. Therefore, this study aimed to evaluate the effect of different mechanical/physical pre-treatments (bead milling, extrusion, freeze-drying, heating, microwave and sonication) combined or not with enzymatic treatments (commercial trypsin and pancreatin) on protein extraction and peptide formation from a *C. vulgaris* suspension. The amount of total protein and peptides released to the supernatant was quantified by Bradford and *o*-phthaldialdehyde assays, respectively. Sodium dodecyl sulphate-polyacrylamide gel electrophoresis was used to analyse the extracted protein fractions. The results showed that extrusion caused a 3-fold increase in total peptides (p < 0.001) compared to no-pretreatment, and trypsin increased peptides formed in bead-milled (p = 0.020) and freeze-dried (p = 0.021) microalga relative to those pre-treatments alone. Some pre-treatments, such as bead milling and microwave, were effective in releasing specific protein fractions, particularly those from 32 to 40 kDa (up to 1.2-fold), compared to control. Pancreatin combined with bead milling decreased 32 to 40 kDa- and 26 kDa-protein fractions (p < 0.010) compared with the sole use of mechanical treatment, whereas the same enzyme mixture associated with microwave produced a similar result for 26 kDa-protein fraction (p = 0.023). Pancreatin also effectively reduced the total protein fraction released after pre-treatment with sonication (p = 0.013). These findings suggest that combining different pre-treatments and enzymatic treatments could improve protein extraction from *C. vulgaris* biomass, providing a useful approach for the development of sustainable protein sources. The present results highlight the need for further studies to assess the efficacy of extrusion in improving the bioaccessibility of *C. vulgaris* proteins in monogastric animals' diets.

## Highlights

• Extrusion pre-treatment caused a 3-fold increase in *Chlorella vulgaris*’ peptides.
• Trypsin increased algal peptides after freeze-drying and bead milling.
• Bead milling and microwave released 32 to 40 kDa-protein fractions.
• Pancreatin reduced 26 kDa-protein fractions after microwave and bead milling.
• Pancreatin could reduce total protein fraction after sonication.

## Graphical abstract

**Figure:**
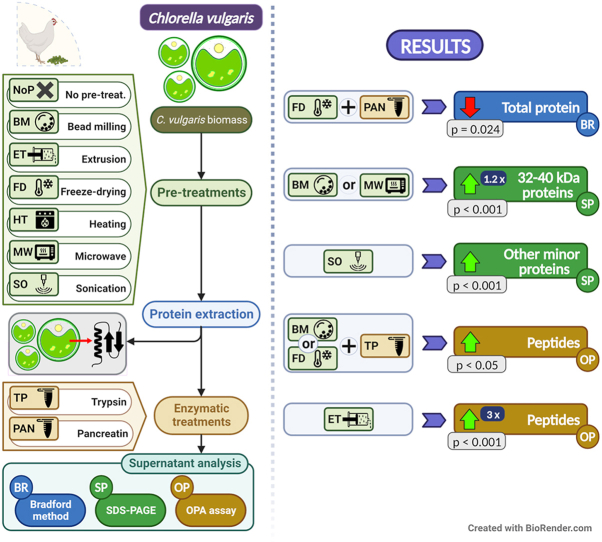

## 1 Introduction

Microalgae, including *Chlorella* species, are recognised for their rapid growth rates and high nutrient content, making them valuable sources for food and feed [1]. *Chlorella* sp., in particular, stands out due to its high-quality protein content, which can constitute up to 67 % of its dry matter. Additionally, *Chlorella* is rich in pigments (such as chlorophylls *a* and *b*, and carotenoids), minerals, and vitamins [2]. These characteristics make it suitable for diverse applications, including human nutrition, cosmetics, animal feed, agriculture and pharmaceuticals [3]. Recent studies have further shown that *Chlorella vulgaris* can enhance biomass productivity by 26 % and lipid productivity by 20 % [4]. Thus, *Chlorella* sp. is a promising sustainable source of essential nutrients and bioactive compounds.

However, the bioavailability of these nutrients is limited by the presence of a recalcitrant cell wall composed primarily of insoluble carbohydrates, such as cellulose and chitin-like polymers [5,6]. The cell wall of *Chlorella* species is highly variable, depending on species, strain, and growth conditions [7]. For example, early-stage *C. vulgaris* cells have a single microfibrillar layer, whereas older cells have a two-layer structure with a thin inner layer and a thicker outer layer divided by an electron-translucent interspace [5]. While the cell wall of *C. vulgaris* lacks a trilaminar matrix, modifications to its composition can occur with ageing towards a more resistant cell wall [8]. Additionally, some *C. vulgaris* proteins form complexes with chlorophyll in thylakoid membranes that are resistant to anionic and non-ionic detergents [8]. These aspects limit the use of *C. vulgaris* as a feed ingredient for monogastric animals, such as poultry and swine. The use of microalgae for industrial purposes is limited by the very small total volume of biomass produced worldwide since the production techniques are not always economically feasible [9]. However, *Chlorella* biomass has also been documented to be beneficial for the removal of vanadium (III) from industrial effluents, highlighting its potential application in bioremediation [10]. An association between biogas production using microalgae followed by the application of the leftover dried biomass for animal feed purposes would optimize their utilization [9].

Protein fractions from *C. vulgaris* exhibit a wide range of molecular weights, varying from 14 to 116 kDa, with their expression being influenced by growth conditions (photoheterotrophic, mixotrophic and autotrophic) [11]. Notably, proteins in photoheterotrophic and mixotrophic cultures had shown higher molecular weights (28–116 kDa) than those in autotrophic cultures (14–23 kDa), with high-density bands at 28 to 34 and 66 kDa, as reported by Piasecka et al. [11], analysing mass spectrometry combined with two-dimensional gel electrophoresis. Among the proteins in photoheterotrophic *C. vulgaris* cultures, those associated with the cytoskeleton (49–77 kDa) or involved in stress response in the chloroplast (71–81 kDa) were identified. Piasecka et al. [11] and Khairy et al. [12] detected two high-density protein bands at 39 and 75 kDa in *C. vulgaris* without characterizing them, using sodium dodecyl sulphate-polyacrylamide gel electrophoresis (SDS-PAGE). Similarly, Tejano et al. [13] identified, with a similar technique, ten predominant proteins in *Chlorella sorokiniana*, ranging from 7.8 to 109 kDa, with some being chloroplast-related proteins such as rubisco (ribulose-1,5-biphosphate carboxylase oxygenase) activase, phosphoglycerate kinase, heat shock proteins and Fe-superoxide dismutase. Despite these findings, the properties of *Chlorella* sp. protein fractions remain inadequately studied.

Mechanical and physical pre-treatments, such as bead milling, high-pressure processing, and ultrasonication, were demonstrated to effectively disrupt the cell wall of *Chlorella* sp. and promote protein extraction. These pre-treatments have significant benefits and limitations. Bead milling, for instance, uses agitation, collision and grinding to disrupt cell walls, making it highly effective for protein extraction. However, it can be energy-intensive and may cause heat generation, potentially degrading sensitive biomolecules [[14], [15], [16], [17]]. High-pressure processing can achieve efficient cell disruption and protein release through the application of extreme pressures, but the required equipment is often costly and may not be suitable for all types of microalgae biomass [14,17,18]. Ultrasonication employs ultrasonic waves to create cavitation bubbles that disrupt cells. While this method is effective and relatively quick, it can result in inconsistent cell disruption and may require optimization for different algal strains [14,[17], [18], [19], [20]]. The suitability of these pre-treatment methods for releasing maximum products from microalgae biomass has been reviewed extensively. For instance, a comprehensive review discusses various pre-treatment methods, highlighting the advantages and disadvantages of each method in the context of microalgae biomass processing [21]. This review emphasizes the importance of selecting appropriate pre-treatment techniques based on the specific requirements of the downstream processes and the type of microalgae used.

Non-mechanical/physical pre-treatments, such as chemical hydrolysis using alkaline solutions and surfactants [15,18,22,23] or carbohydrate-active enzymes, including lysozyme, chitinase, cellulase and trypsin [19,20,24,25], were shown to be effective in degrading the *Chlorella* sp. cell wall and promoting protein extraction. Several studies have evaluated the impact of enzymatic activity on crude protein and dry-weight digestibility of *Chlorella* sp. *In vitro* tests with pepsin and pancreatin have shown digestibility coefficients above 70 % [26,27]. Other studies have investigated the combination of peptidases with other non-mechanical pre-treatments [23] or carbohydrases [28] for the hydrolysis of *C. vulgaris* cell wall polysaccharides and glycoproteins.

In previous studies, some promising results have been reported for the protein extraction of *Chlorella* sp., such as the combination of a low-temperature and high-pressure procedure with proteases (papain, trypsin and alcalase) [19], bead milling with a commercial *Bacillus licheniformis* protease [29], or ultrasonication and homogenization with a cellulase [20]. However, while the effectiveness of some individual mechanical/physical or enzymatic pre-treatments on protein extraction from *Chlorella* sp. has already been investigated, few have explored the potential synergistic effects of combining these treatments. The previous studies did not compare several pre-treatments, followed or not by an enzymatic treatment, as the present study aims to accomplish. The report by Wang et al. [19] used a low-temperature and high-pressure procedure, but, herein, the extrusion method will be applied instead. Moreover, the protease used by Alavijeh et al. [29] was different from the commercial trypsin or pancreatin considered in the present study, which are advantageous for an industrial application in monogastric animal feed. In addition, Zhang et al. [20] applied a cellulase to disrupt the microalga cell wall without assessing the effect of any protease in the hydrolysis of microalga proteins. Moreover, to the best of our knowledge, the present study is the first to analyse the impact of a combination of such pre-treatments on the production and releasement of total peptides from *Chlorella* sp. biomass and on the hydrolysis of major algal protein fractions, as well as the influence of extrusion or microwave combined with peptidases on protein extraction. Altogether, these aspects highlight the novelty of the present study in comparison to other reports. The explanation of all pre-treatments is exploited in Spínola et al. [30]. A recent *in vitro* study [31] by our team showed a decreased solubility of total protein from *C. vulgaris* treated with extrusion, which was suggested to increase protein susceptibility to enzymatic hydrolysis, and, thus, reinforcing the importance of the current experiment. Therefore, the present study aimed to evaluate the impact of mechanical/physical pre-treatments (bead milling, extrusion, freeze-drying, heating, microwave and sonication) combined with trypsin (EC 3.4.21.4) or pancreatin (pancreatic peptidase, lipase, and amylase) on protein extraction and peptide production from *C. vulgaris* biomass. The present results are expected to help to fill the gap that still exists in terms of enhancing *C. vulgaris* protein bioaccessibility in monogastric animals' diets. The analysis of algal protein bioaccessibility was done by quantifying total soluble protein content, using the Bradford method, and determining protein fraction profile, with SDS-PAGE. The formation of peptides was evaluated using *o*-phthaldialdehyde assay.

## 2 Material and methods

### 2.1 Chlorella vulgaris composition and mechanical/physical pre-treatments

The dried powder of *Chlorella vulgaris* (Allmicroalgae Natural Products SA, Pataias, Portugal) was produced in heterotrophic cultures. The granulometry of the powder was less than 63 μm, verified using a woven wire mesh sieve (Retsch GmbH, Haan, Germany). Table 1 presents the chemical composition of *C. vulgaris* provided by Allmicroalgae. The results were obtained using routine methods [32], and the pigment composition was analysed using the method described by Ritchie [33], as previously reported [30,34].

**Table 1:** Proximal composition of Chlorella vulgaris (mean values provided by Allmicroalgae company).

Composition in nutrients	
Energy (MJ/kg)	15.5
Moisture (%)	4.00
Ash (% dry matter)	4.48
Crude carbohydrates (% dry matter)	36.3
Crude fat (% dry matter)	8.44
Crude fibre (% dry matter)	19.6
Crude protein (% dry matter)	31.3
**Composition in pigments**	
Chlorophyll (% dry matter)	0.60
Total carotenoids (% dry matter)	0.16

The microalgae were treated with six pre-treatments: bead milling, extrusion, freeze-drying, heating, microwave and sonication, according to Costa et al. [34] and Spínola et al. [30]. Briefly, bead milling was done by mixing the algal suspension (20 g/L in Phosphate Buffered Saline (PBS)) with 0.5 mm zirconium beads (1 bead per 1 mL) for 30 min at 2000 rpm in a shaking orbit (Multi Reax Heidolph Instruments, Schwabach, Germany). The extrusion method of microalga powder was performed in an extruder from Sparos company (Olhão, Algarve, Portugal) as previously reported [30,34], but using a lower temperature (114 °C instead of 118 °C). Microalgal suspension at 20 g/L was done after this procedure. The freeze-drying consisted of algal powder lyophilisation (Labogene, CoolSafe, Frilabo, Milheirós, Portugal) for 24 h, after overnight freezing at −80 °C, and then the freeze-dried microalga was suspended in PBS. The heating was performed by exposing the powder alga at 70 °C for 30 min in a stove (Melag, Geneststraβe, Berlin, Germany), followed by resuspension in PBS solution. The microwave method was done in a microwave (Whirlpool, Household Microwave Oven, MI, USA) under the keep warm program until the microalga suspension at 20 g/L was boiling. The sonication was carried out in an ultrasound device using a probe for ultrasonic wave diffusion (Bandelin ultrasonic homogenizer, Heinrichstraβe, Berlin, Germany) and seven cycles at 200 W, 20 kHz and 70 % potency for 15 min. Table 2 summarizes the conditions of the six mechanical/physical pre-treatments.

**Table 2:** Summary of the six mechanical/physical pre-treatment conditions.

Pre-treatments	Conditions
Bead milling	Algal suspension with 0.5 mm zirconium beads (1 bead per 1 mL) for 30 min at 2000 rpm in a shaking orbit.
Extrusion	Microalgae at 34 bars and 114 °C for 3–7 s, followed by water addition at 340 mL/min and drying at 120 °C for 8–10 min.
Freeze-drying	Algal biomass' lyophilisation for 24 h, after overnight freezing at −80 °C.
Heating	Exposing dried microalgae at 70 °C for 30 min on a stove.
Microwave	Keep warm program until microalga suspension is boiling.
Sonication	Ultrasound device regulated for seven cycles at 70 % potency, 200 W, 20 kHz, for 15 min.

### 2.2 Incubation of pre-treated Chlorella vulgaris with enzymes

The incubation assay was performed in a 24-well microplate with five replicates per treatment (n = 5). Each enzyme (pancreatin at 350 FIP-U/g protease, 6000 FIP-U/g lipase, 7500 FIP-U/g amylase, and trypsin type II-S at 1000–2000 units/mg dry weight) was added at a concentration of 20 μg/mL to the pre-treated algal suspension or an equivalent volume of PBS as a control. The enzyme-substrate ratio was maintained at 1:1000 for all treatments. The microplate was incubated at 37 °C and 160 rpm for 16 h and then centrifuged for 15 min at 1500×*g* to recover 1 mL of supernatants. The enzymes used were pancreatin (350 FIP-U/g protease, 6000 FIP-U/g lipase, 7500 FIP-U/g amylase; Merck, Darmstadt, Germany) and trypsin (type II-S, 1000–2000 units/mg dry weight, Sigma-Aldrich, St Louis, MO, USA) dried powders from porcine pancreas.

### 2.3 Quantification of protein fractions

The protein fraction profile was obtained by SDS-PAGE electrophoresis, as previously described [30,34], but with some modifications. Succinctly, 8 μL of each replicate was loaded into 12 % PAGE gels, as well as 5 μL of low molecular weight (about 9.00 μg of protein) (LMW) protein marker (18.5–96 KDa) (Nzytech, Lisbon, Portugal). The gel images were scanned and the three most prominent protein fractions (fraction 1, 66–96 kDa; fraction 2, 32–40 kDa; and fraction 3, 26 kDa) were selected, together with other protein fractions, for quantification of their relative densities using Image J software (NIH, Bethesda, MA, USA). Fraction 1 might have corresponded to proteins associated with the cytoskeleton or located in the chloroplast for stress response (*e.g*., heat shock proteins), whereas fractions 2 and 3 possibly contained unidentified chloroplast-related proteins among other proteins [11,13].

### 2.4 Determination of coefficient of protein degradation

The coefficient of protein degradation (CPD) for each quantified protein fraction was calculated according to Costa et al. [34], as an adaptation to that described in previous studies [35,36]. Briefly, CPD values, which were expressed as percentages, allowed the assessment of protein hydrolysis extent and translated the relation between the reduction in protein optical density due to enzymatic hydrolysis and the protein optical density before hydrolysis.

### 2.5 Assessment of total soluble protein using the Bradford method

Total solubilized protein was determined, in the supernatant, spectrophotometrically with the Bradford method [37], as previously described [30,34]. Indeed, 1.5 mL of Bradford solution (PanReac, AppliChem, ITW Reagents, Darmstadt, Germany) was mixed with 30 μL of the supernatant sample. The absorbance was read at 595 nm using a spectrophotometer UV–Vis (Genesys 40/50, ThermoFisher scientific, MA, USA), after 5 min at room temperature. The same standard curve, using bovine serum albumin (BSA) (Sigma-Aldrich, MO, USA) at concentrations from 0.0125 to 1 mg/mL as described by Costa et al. [34] and Spínola et al. [30], was applied for protein quantification. The extraction yield of total water-soluble protein in the supernatant was determined according to an adaptation of Postma et al. [14]:

Protein extraction yield (%) = 100 × [CpTRAT/CpBiomass - CpCON/CpBiomass]

Where: CpTRAT = concentration of protein (g/L) obtained with pre-treatments; CpCON = concentration of protein (g/L) obtained with control; CpBiomass = concentration of protein (g/L) in total biomass.

The concentration of protein in total biomass (6 g/L) was calculated considering 30.0 % dry weight of crude protein and 20 g/L of *C. vulgaris* concentration after resuspension in PBS buffer.

### 2.6 Quantification of total peptides using o-phthaldialdehyde assay

Free amino acids and peptides were determined by spectrophotometry with *o*-phthaldialdehyde (OPA) assay, as reported by Sedighi et al. [38] and Vizcaíno et al. [36], and adapted by Costa et al. [34] and Spínola et al. [30]. In addition, the standard curve was the same as previously described [3,34]. Briefly, 200 μL of sample was added to 100 μL of 20 % trichloroacetic acid and the mixture was centrifuged at 12,000 g for 15 min. Afterwards, 200 μL of supernatant was mixed with 1 mL of daily prepared OPA, according to Sedighi et al. [38] and Vizcaíno et al. [36], and incubated at room temperature for 5 min. Then, the absorbance was determined at 340 nm, and peptone (5–500 mg/L) was used for the standard curve.

### 2.7 Data statistical analysis

ANOVA and Tukey–Kramer methods (PDIFF option) were performed for multiple comparisons of adjusted least square means using general linear models of Statistical Analysis System (SAS) software (SAS Institute Inc., Cary, NC). Levene's test was applied to test variance homogeneity. *P*-values were considered significant for α = 0.05.

## 3 Results and discussion

### 3.1 Protein (Bradford method) and peptide (OPA assay) concentrations from Chlorella vulgaris influenced by mechanical/physical pre-treatments and enzymatic treatment

The effect of various treatments on the total protein and peptide concentrations in *C. vulgaris* is presented in Table 3. Overall, the results indicate that pancreatin significantly decreased protein content in freeze-dried microalga (*p* = 0.024), while extrusion was the only pre-treatment to significantly increase total peptide concentration, achieving an almost 3-fold increase (*p* < 0.001).

**Table 3:** Influence of mechanical/physical pre-treatments, either alone or combined with enzymes, on the concentration of total soluble protein (g/L) (Bradford method) and peptides (mg/L) (OPA assay) in the supernatant fraction of *Chlorella vulgaris* (n = 5).

Item	Enzymes	Pre-treatments^a^							SEM^b^	*p*-value
		NoP	BM	ET	FD	HT	MW	SO		
**Total protein (g/L)**	No enzyme	0.08^AB^	0.12	0.06	0.09^A^	0.06^AB^	0.10	0.09	0.030	0.698
	Trypsin	0.10^A^	0.15	0.05	0.10^A^	0.10^A^	0.13	0.11	–	–
	Pancreatin	0.01^B^	0.10	0.02	0.02^B^	0.02^B^	0.10	0.06	–	–
	SEM	0.017	0.050	0.014	0.019	0.017	0.056	0.016	–	–
	*P*-value	0.013	0.794	0.198	0.024	0.017	0.934	0.068	–	–
**Total peptides (mg/L)**	No enzyme	13.1^b^	16.8^bB^	32.4^a^	11.2^bB^	12.4^bAB^	14.2^b^	19.1^b^	1.81	<0.001
	Trypsin	16.5	24.6^A^	31.6	17.7^A^	18.1^A^	16.2	21.5	–	–
	Pancreatin	12.9	17.6^B^	31.4	14.6^AB^	11.7^B^	10.9	15.8	–	–
	SEM	1.68	1.82	2.36	1.39	1.62	2.13	1.73	–	–
	*P*-value	0.271	0.020	0.952	0.021	0.030	0.249	0.103	–	–
**Total protein extraction yield (%)**		–	0.75	−0.36	0.30	−0.27	0.43	0.15	0.673	0.845

^a,b^ Different lowercase superscripts indicate significant differences between pre-treatments (*p* < 0.05).

^A, B^ Different uppercase superscripts indicate significant enzyme effects (*p* < 0.05).

a No pre-treatment (NoP); bead milling (BM); extrusion (ET); freeze-drying (FD); heating (HT); microwave (MO); sonication (SO).

b Standard error of the mean.

Trypsin was effective in increasing soluble peptide concentrations (*p* < 0.050) in bead-milled and freeze-dried microalga compared to non-enzymatic treatments. Although there was a non-significant enhancement of peptides in heated *C. vulgaris* treated with trypsin, pancreatin showed intermediate peptide release from freeze-dried microalga. The maximum protein concentration released into the supernatant was 0.13 g/L (0.68 % dry matter; 2.17 % total protein), significantly lower than previously reported values [14].

The significant increase in peptide concentrations, rather than total proteins, suggests that extrusion (combining high pressure and temperature) may enhance peptide formation by disrupting the cell wall and promoting protein hydrolysis. Previous studies have demonstrated the effectiveness of high-pressure treatments in protein extraction, achieving yields of up to 76 % from freeze-thawed *C. vulgaris*. However, our study uniquely shows that extrusion can specifically increase peptide concentrations, an aspect not extensively explored previously.

The use of freeze-drying alone did not significantly enhance protein extraction but did so when combined with pancreatin, suggesting a synergistic effect where the pre-treatment makes the cell wall more permeable to enzyme action. This observation aligns with Wang and Zhang [19], who reported increased protein extraction when freeze-thawing was combined with enzymatic hydrolysis.

Bead milling effectively disrupted the *C. vulgaris* cell wall, facilitating trypsin's action and increasing peptide formation. This mechanical pre-treatment likely enhances the accessibility of proteins to enzymatic hydrolysis, consistent with previous findings [14,17].

While the total soluble protein extraction yield did not vary significantly among treatments (*p* = 0.845), pre-treatments like bead milling, freeze-drying, microwave, and sonication showed a numerical increase in protein yield. Bead milling achieved up to 96 % protein extraction from freeze-dried microalgae, highlighting its effectiveness in cell disruption [14].

### 3.2 Protein concentration quantified in SDS-PAGE gel of Chlorella vulgaris affected by mechanical/physical pre-treatments and enzymatic treatment

Table 4 and Fig. 1(A and B) show the effects of treatments on the concentration of total protein and specific protein fractions in *C. vulgaris*. Bead milling and microwave treatments significantly increased the amount of the 32–40 kDa protein fraction (F2) (*p* < 0.001), while sonication increased other protein fractions but decreased the 26 kDa fraction (F3) (*p* < 0.001).

**Table 4:** Influence of mechanical/physical pre-treatments, either alone or combined with enzymes, on the concentration of total protein and protein fractions (g/L) (SDS-PAGE method) of *Chlorella vulgaris* supernatant (n = 5).

Item	Enzymes	Pre-treatments^a^							SEM^b^	*p*-value
		NoP	BM	ET	FD	HT	MW	SO		
**Protein fraction (66–96 kDa)**	No enzyme	1.28^abB^	1.50^aA^	1.26^ab^	1.24^b^	1.19^bB^	1.50^a^	1.15^b^	0.054	<0.001
	Trypsin	1.14^C^	1.47^AB^	1.15	1.23	1.13^B^	1.48	1.08	–	–
	Pancreatin	1.47^A^	1.33^B^	1.37	1.30	1.41^A^	1.38	1.05	–	–
	SEM	0.033	0.042	0.073	0.023	0.037	0.060	0.030	–	–
	*P*-value	<0.001	0.028	0.160	0.085	<0.001	0.379	0.096	–	–
**Protein fraction (32–40 kDa)**	No enzyme	1.26^bB^	1.51^aA^	1.27^b^	1.18^b^	1.18^bAB^	1.48^a^	1.17^b^	0.046	<0.001
	Trypsin	1.16^C^	1.51^A^	1.16	1.20	1.11^B^	1.49	1.13	–	–
	Pancreatin	1.38^A^	1.28^B^	1.29	1.23	1.30^A^	1.30	1.04	–	–
	SEM	0.023	0.045	0.052	0.043	0.049	0.054	0.034	–	–
	*P*-value	<0.001	0.004	0.208	0.731	0.052	0.051	0.059	–	–
**Protein fraction (26 kDa)**	No enzyme	1.39^ab^	1.50^aA^	1.28^abc^	1.32^abc^	1.20^bcA^	1.50^aA^	1.13^cAB^	0.052	<0.001
	Trypsin	1.34	1.53^A^	1.24	1.41	1.15^A^	1.51^A^	1.16^A^	–	–
	Pancreatin	1.39	1.29^B^	1.30	1.22	1.06^B^	1.33^B^	1.05^B^	–	–
	SEM	0.028	0.040	0.044	0.082	0.024	0.045	0.025	–	–
	*P*-value	0.350	0.003	0.601	0.312	0.004	0.023	0.030	–	–
**Other proteins**	No enzyme	2.73^b^	2.93^b^	3.02^b^	2.99^b^	3.23^b^	2.94^b^	3.88^aA^	0.142	<0.001
	Trypsin	2.74	2.93	2.83	2.89	3.10	3.10	3.68^AB^	–	–
	Pancreatin	3.01	2.87	2.78	3.09	3.38	2.96	2.80^B^	–	–
	SEM	0.147	0.115	0.178	0.067	0.124	0.122	0.241	–	–
	*P*-value	0.333	0.905	0.614	0.143	0.329	0.627	0.019	–	–
**Total protein**	No enzyme	6.66^B^	7.43^A^	6.83^A^	6.73	6.79	7.42	7.33^A^	0.185	0.010
	Trypsin	6.38^B^	7.44^A^	6.38^B^	6.72	6.50	7.58	7.04^A^	–	–
	Pancreatin	7.25^A^	6.76^B^	6.73^AB^	6.84	7.14	6.97	5.95^B^	–	–
	SEM	0.136	0.162	0.114	0.183	0.197	0.243	0.288	–	–
	*P*-value	0.002	0.018	0.040	0.876	0.109	0.231	0.013	–	–

^a,b,c^ Different lowercase superscripts indicate significant differences between pre-treatments (*p* < 0.05).

^A, B,C^ Different uppercase superscripts indicate significant enzyme effects (*p* < 0.05).

a No pre-treatment (NoP); bead milling (BM); extrusion (ET); freeze-drying (FD); heating (HT); microwave (MO); sonication (SO).

b Standard error of the mean.

**Fig. 1:**
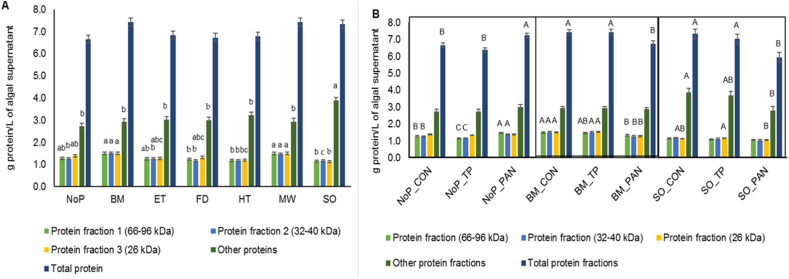
(A,B). Impact of mechanical/physical pre-treatments (**A**) and enzymes (**B**) on the amount of protein fractions quantified in the supernatant of *Chlorella vulgaris* loaded in SDS-PAGE gels (*n* = 5): no pre-treatment (NoP); bead milling (BM); extrusion (ET); freeze-drying (FD); heating (HT); microwave (MO); sonication (SO); control (CON); trypsin (TP); pancreatin (PAN). ^a,b,c^ Different lowercase superscripts indicate significant differences among pre-treatments for each fraction (*p* < 0.05). ^A,B^ Different uppercase superscripts indicate significant differences for each fraction obtained with no pre-treatment, bead milling or heating alone or combined with enzymes (*p* < 0.05). Bars indicate the standard error of the mean.

Trypsin reduced the 66–96 kDa protein fraction (F1) in the supernatant of non-pre-treated microalga, whereas pancreatin had the opposite effect (*p* < 0.001). Pancreatin increased F1 after heating but reduced it after bead milling (*p* = 0.028). The enzymatic treatments generally led to the degradation of F3 after various pre-treatments, indicating effective protein hydrolysis.

The isolated use of bead milling effectively released chloroplast-related proteins, as evidenced by the increased F2, consistent with the release of intra-thylakoid pigments and rubisco-derived protein fractions reported by Safi et al. [15] and Postma et al. [16]. This suggests that bead milling disrupts the thylakoid membrane, facilitating protein release.

Microwave treatment's effectiveness in releasing F2 proteins from *C. vulgaris* biomass is attributed to the combined effects of heating and electroporation, which disrupt cell walls, as reported by Chew et al. [39]. This method has shown high protein recovery rates, demonstrating its potential for efficient protein extraction.

Sonication, despite variable protein yield outcomes, can effectively release less abundant proteins, though its efficiency may be compromised in freeze-dried cells due to aggregation, reducing cell dispersion and ultrasonic wave impact [40].

The reduction of total protein fractions due to combining mechanical pre-treatments with enzymatic treatments (e.g., bead milling with pancreatin or extrusion with trypsin) underscores the synergy between physical disruption and enzymatic hydrolysis. This combination facilitates more complete protein breakdown, as supported by previous studies [19,29].

### 3.3 Degradation of proteins in trypsin-pre-treated Chlorella vulgaris

The coefficients of protein degradation (CPD) due to trypsin are shown in Table 5 and Fig. 2(A–D). Trypsin's effect on F1 was not significantly different between treatments (p = 0.440), but numerical hydrolysis was observed, particularly in non-pre-treated microalga. Trypsin tended to increase CPD values for F3 in no-pre-treated, heated or extruded *C. vulgaris* (p = 0.050).

**Table 5:** Influence of trypsin or pancreatin on coefficients of protein degradation (CPD) (%) of *Chlorella vulgaris* protein fractions in biomass treated with several mechanical/physical pre-treatments (n = 5).

Item	Enzymes	Pre-treatments^a^							SEM^2^	*p*-value
		NoP	BM	ET	FD	HT	MW	SO		
**Protein fraction (66–96 kDa)**	Trypsin	10.1	1.63	6.88	1.13	4.19	1.86	5.85	1.244	0.440
	Pancreatin	−5.24	−3.32	0.33	−0.86	−4.66	−5.70	−0.83	1.096	0.682
**Protein fraction (32–40 kDa)**	Trypsin	8.26	0.01	7.90	−2.12	5.13	−0.41	3.05	1.292	0.175
	Pancreatin	−2.40	3.06	4.80	5.00	2.48	−3.74	6.95	1.237	0.175
**Protein fraction (26 kDa)**	Trypsin	3.83	−2.22	2.96	−7.13	3.38	−0.74	−2.52	1.054	0.050
	Pancreatin	−3.11^b^	2.00^b^	5.13^b^	2.70^b^	18.4^a^	−4.10^b^	3.56^b^	1.408^b^	<0.001
**Other proteins**	Trypsin	−1.06^ab^	0.01^ab^	6.59^a^	3.17^ab^	3.87^ab^	−5.29^b^	5.69^ab^	1.126	0.045
	Pancreatin	−3.54	0.50	0.97	0.08	−7.18	−0.38	7.61	1.303	0.094
**Total protein**	Trypsin	4.24	−0.08	6.36	0.07	4.07	−1.91	4.04	0.893	0.117
	Pancreatin	−3.65	0.52	2.40	1.44	−0.01	−2.63	5.52	0.908	0.113

^a,b^ Significant differences among pre-treatments for each fraction are indicated by different superscripts (p < 0.05).

CPD = (OD1 − OD2)/OD1 × 100; OD1 = optical density of each protein band before hydrolysis, OD2 = optical density of each protein band after hydrolysis. The optical densities were determined using Image J software (NIH, Bethesda, MA, USA).

a NoP, no pre-treatment BM, bead milling; ET, extrusion; FD, freeze-drying; HT, heating; MO, microwave; SO, sonication. 2 Standard error of the mean.

**Fig. 2:**
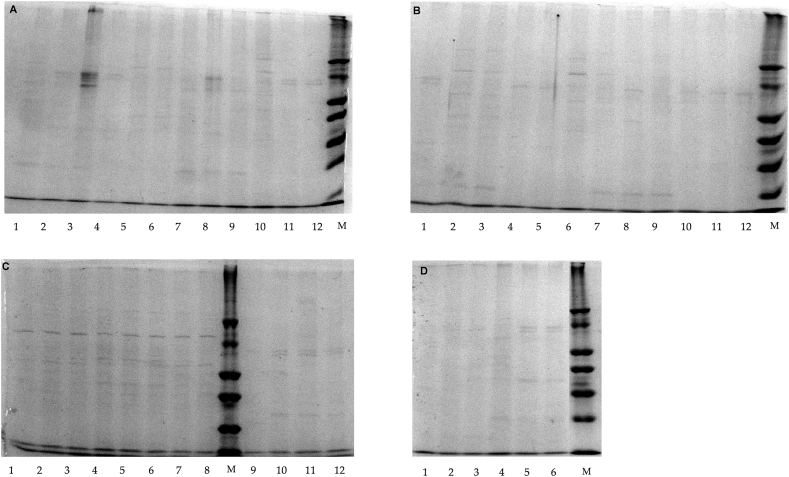
(A–D). Sodium dodecyl-sulphate polyacrylamide gel electrophoresis (SDS-PAGE) representing the effect of trypsin on the hydrolysis of *Chlorella vulgaris* proteins after pre-treatment (*n* = 3). Gel A: microwave (1–3, trypsin; 4–6, control) and bead milling (7–9, trypsin; 10–12, control); gel B: heating (1–3, trypsin; 4–6, control) and extrusion (7–9, trypsin; 10–12, control); gel C: freeze-drying (1–3, control; 4–6, trypsin) and no pre-treatment (7–9, control; 10–12, trypsin); gel D: sonication (1–3, control; 4–6, trypsin). M, low molecular weight protein marker.

The slight increase in CPD after extrusion followed by trypsin treatment is consistent with Wang and Zhang [19], who reported mild hydrolysis of proteins with similar pre-treatments. The variability between replicates and lack of initial protein substrate explain the subtle effects observed.

### 3.4 Degradation of proteins in pancreatin-pre-treated Chlorella vulgaris

The effect of pancreatin on the coefficients of protein degradation (CPD) of non- or pre-treated *C. vulgaris* is presented in Table 5, and the most representative SDS-PAGE gels for protein hydrolysis by pancreatin, after microalga pre-treatments, are shown in Fig. 3(A–D). Although several *p*-values are greater than 0.05, indicating that not all differences are statistically significant, there are still meaningful trends and significant observations worth discussing.

**Fig. 3:**
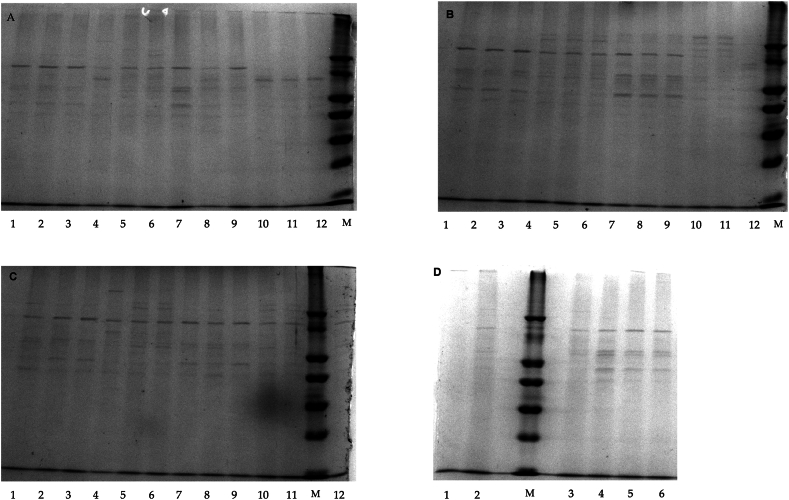
(A–D). Sodium dodecyl-sulphate polyacrylamide gel electrophoresis (SDS-PAGE) representing the effect of pancreatin on the hydrolysis of *Chlorella vulgaris* proteins after pre-treatment (*n* = 3). Gel A: microwave (1–3, pancreatin; 4–6, control) and bead milling (7–9, pancreatin; 10–12, control); gel B: heating (1–3, pancreatin; 4–6, control) and extrusion (7–9, pancreatin; 10–12, control); gel C: no pre-treatment (1–3, pancreatin; 4–6, control) and freeze-drying (7–9, pancreatin; 10–12, control); gel D: sonication (1–3, control; 4–6, pancreatin). M, low molecular weight protein marker.

For instance, heating combined with pancreatin significantly increased the degradation of the 26 kDa protein fraction (F3) compared to other treatments (*p* < 0.001), demonstrating the effectiveness of this combination in targeting low molecular weight proteins. This is consistent with previous studies indicating that heating can enhance the accessibility of proteins to enzymatic hydrolysis by denaturing protein structures and disrupting cell walls [41,42].

Furthermore, while the effects of pancreatin on the degradation of the 66–96 kDa and 32–40 kDa protein fractions (F1 and F2) were not statistically significant (*p* > 0.05), the numerical trends suggest potential enhancements in protein hydrolysis that could be further explored with larger sample sizes or additional replicates. The higher CPD values for other proteins in sonicated *C. vulgaris* also point to a possible synergistic effect of sonication and pancreatin, which may warrant further investigation despite the lack of statistical significance (*p* = 0.094).

Similarly, trypsin's effect on protein degradation showed some significant results, such as the reduction in the other protein fractions (*p* = 0.045) and a trend towards increased CPD values for the 26 kDa fraction (*p* = 0.050). These results suggest that trypsin can be effective in hydrolysing certain protein fractions, especially when combined with specific pre-treatments like extrusion and heating.

## 4 Conclusion

Overall, the mechanical/physical pre-treatments under these experimental conditions did not promote microalga protein extraction. However, the present report highlights the impact of the extrusion process, which is barely known for its ability to extract protein from *C. vulgaris* biomass, on the formation of total algal peptides leading to a 3-fold increase of these compounds. In addition, other pre-treatments increased the susceptibility of total protein to trypsin and pancreatin hydrolysis with consequent peptide production. Indeed, the amount of soluble protein in algal supernatant decreased with freeze-drying associated with pancreatin, possibly due to protein conversion into peptides, whereas total peptides increased with freeze-drying or bead milling combined with trypsin. The combination of pre-treatments with enzymatic treatments could also promote the hydrolysis of major protein fractions detected in SDS-PAGE gel. Bead milling combined with pancreatin hydrolysed and decreased F1, F2, and F3 protein fractions, whereas bead milling and sonication associated with pancreatin led to a reduction of total protein quantified in the gel.

These findings suggest that the pre-treatments could promote protein extraction and increase their susceptibility to enzymatic activity, which shows their potential application to treat *C. vulgaris* for the feed industry. Despite that, the present study has some limitations, which include the low protein extraction yield obtained with the pre-treatments evidencing the recalcitrance of *C. vulgaris* cell wall, the low number of samples analysed due to the constraint of performing several *in vitro* incubations in parallel, and the difficulties of applying most of the pre-treatments at an industrial level. Nevertheless, the extrusion method could significantly increase total microalga peptides and can be easily scaled up to an industrial level. An increase in such nutritive and bioactive compounds could have benefits in terms of growth performance in monogastric animals. Indeed, a recent study showed the importance of extrusion pre-treatment in alleviating the deleterious impact on the broiler's growth by feeding 15 % of *A. platensis* [49]. Therefore, although the total protein yield is low and further research is still required to improve protein extraction from *C. vulgaris* biomass with the pre-treatments, *in vivo* studies will be developed by our team to assess the efficacy of extrusion to improve *C. vulgaris* protein bioaccessibility in microalga supplemented broiler diets.

## Data availability statement

All data generated or analysed during this study are included in this published article.

## Additional information

No additional information is available for this paper.

## CRediT authorship contribution statement

**Mónica Mendes Costa:** Writing – original draft, Investigation, Formal analysis, Data curation. **Maria Pinheiro Spínola:** Writing – original draft, Investigation, Formal analysis, Data curation. **Victor Diogo Alves:** Visualization. **José António Mestre Prates:** Writing – review & editing, Project administration, Funding acquisition, Conceptualization.

## Declaration of competing interest

The authors declare that they have no known competing financial interests or personal relationships that could have appeared to influence the work reported in this paper.

## Figures and Tables

**figs1:**
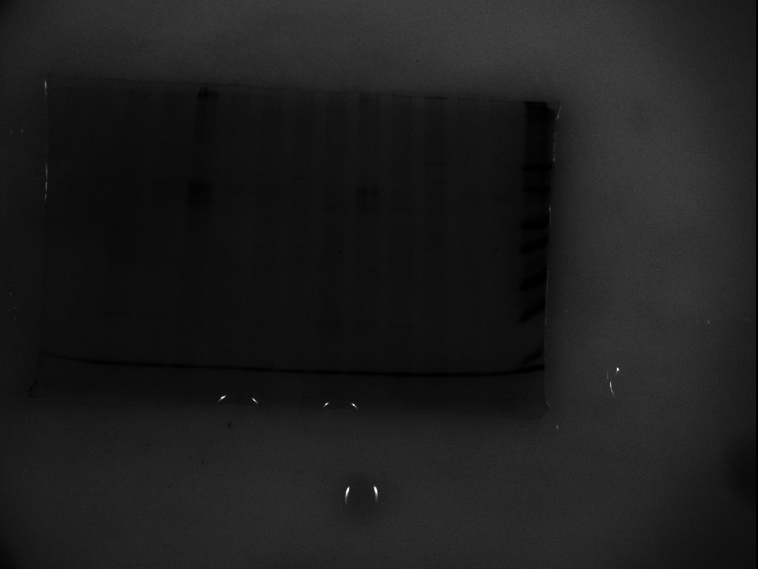


**figs2:**
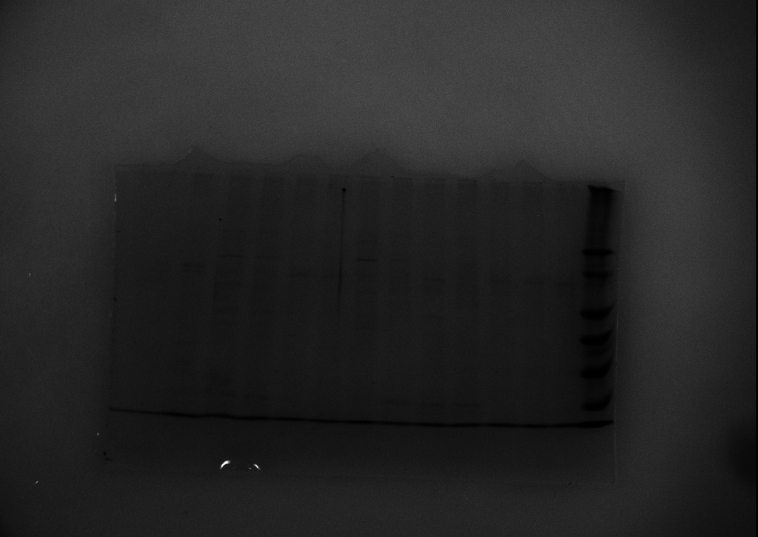


**figs3:**
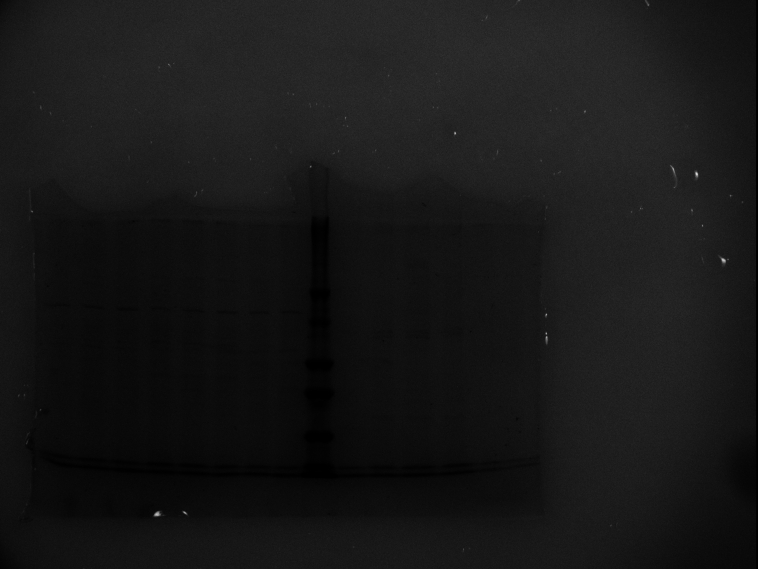


**figs4:**
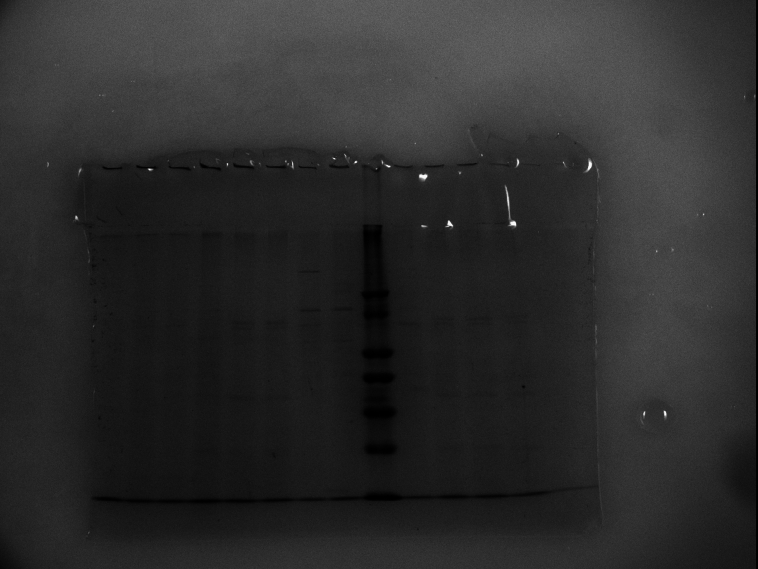


**figs5:**
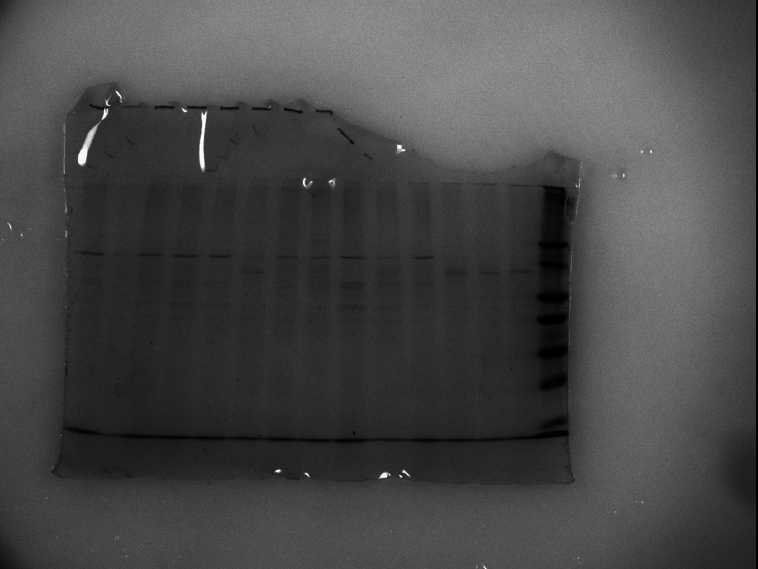


**figs6:**
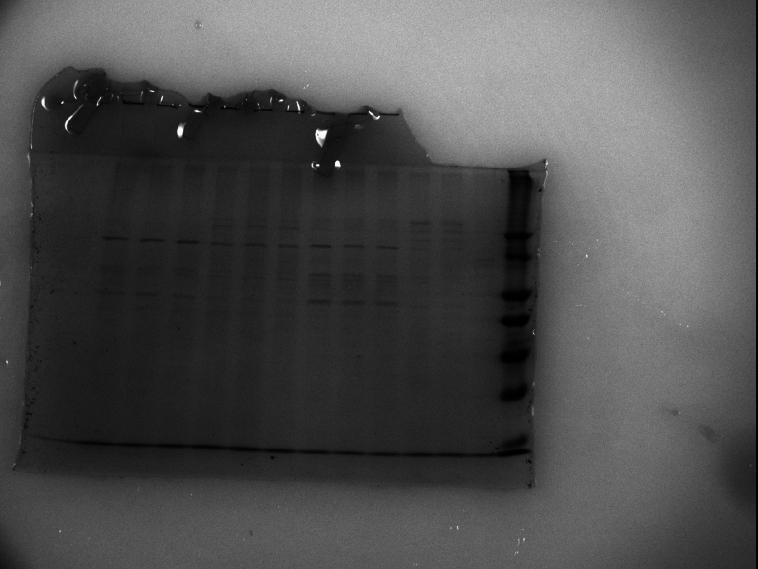


**figs7:**
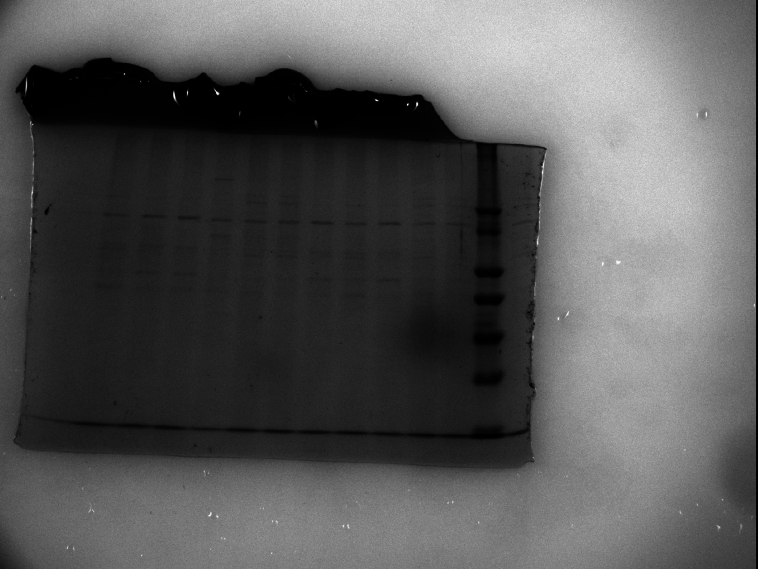


**figs8:**